# Preoperative Chemotherapy for Operable Colon Cancer: Mature Results of an International Randomized Controlled Trial

**DOI:** 10.1200/JCO.22.00046

**Published:** 2023-01-19

**Authors:** Dion Morton, Matthew Seymour, Laura Magill, Kelly Handley, James Glasbey, Bengt Glimelius, Andy Palmer, Jenny Seligmann, Søren Laurberg, Keigo Murakami, Nick West, Philip Quirke, Richard Gray

**Affiliations:** ^1^ University Hospital Birmingham, Birmingham, United Kingdom; ^2^ St James's University Hospital, Leeds, United Kingdom; ^3^ University of Birmingham Clinical Trials Unit, Birmingham, United Kingdom; ^4^ Uppsala University, Uppsala, Sweden; ^5^ Aarhus University, Aarhus, Denmark; ^6^ Division of Pathology and Data Analytics, School of Medicine, University of Leeds, Leeds, United Kingdom; ^7^ Nuffield Department of Population Health, University of Oxford, Oxford, United Kingdom

## Abstract

Neoadjuvant chemotherapy (NAC) has potential advantages over standard postoperative chemotherapy for locally advanced colon cancer but requires formal evaluation.

**METHODS:**

Patients with radiologically staged T3-4, N0-2, M0 colon cancer were randomly allocated (2:1) to 6 weeks oxaliplatin-fluoropyrimidine preoperatively plus 18 postoperatively (NAC group) or 24 weeks postoperatively (control group). Patients with *RAS*-wildtype tumors could also be randomly assigned 1:1 to receive panitumumab or not during NAC. The primary end point was residual disease or recurrence within 2 years. Secondary outcomes included surgical morbidity, histopathologic stage, regression grade, completeness of resection, and cause-specific mortality. Log-rank analyses were by intention-to-treat.

**RESULTS:**

Of 699 patients allocated to NAC, 674 (96%) started and 606 (87%) completed NAC. In total, 686 of 699 (98.1%) NAC patients and 351 of 354 (99.2%) control patients underwent surgery. Thirty patients (4.3%) allocated to NAC developed obstructive symptoms requiring expedited surgery, but there were fewer serious postoperative complications with NAC than with control. NAC produced marked T and N downstaging and histologic tumor regression (all *P* < .001). Resection was more often histopathologically complete: 94% (648/686) versus 89% (311/351), *P* < .001. Fewer NAC than control patients had residual or recurrent disease within 2 years (16.9% [118/699] *v* 21.5% [76/354]; rate ratio, 0.72 [95% CI, 0.54 to 0.98]; *P* = .037). Tumor regression correlated strongly with freedom from recurrence. Panitumumab did not enhance the benefit from NAC. Little benefit from NAC was seen in mismatch repair–deficient tumors.

**CONCLUSION:**

Six weeks of preoperative oxaliplatin-fluoropyrimidine chemotherapy for operable colon cancer can be delivered safely, without increasing perioperative morbidity. This chemotherapy regimen, when given preoperatively, produces marked histopathologic down-staging, fewer incomplete resections, and better 2-year disease control. Histologic regression after NAC is a strong predictor of lower postoperative recurrence risk so has potential use as a guide for postoperative therapy. Six weeks of NAC should be considered as a treatment option for locally advanced colon cancer.

## INTRODUCTION

Colorectal cancer is the second commonest cancer worldwide, with 1.7 million diagnoses annually.^1^ Standard treatment is surgery followed by adjuvant oxaliplatin-fluoropyrimidine chemotherapy for those with a moderate- to high-risk disease.^2^ Despite adjuvant chemotherapy (AC), 20%-30% of patients develop recurrent disease that is usually incurable.^3,4^ Preoperative or neoadjuvant chemotherapy (NAC) has substantially improved outcomes in other gastrointestinal cancers^5,6^ and has potential advantages over postoperative AC in colon cancer. Shrinking tumors before surgery may reduce the risk of incomplete resection and tumor cell-shedding during surgery.^7^ NAC can start many weeks earlier than AC so could be more effective in eradicating micrometastases,^8^ particularly as surgery induces growth factor activity, potentially stimulating tumor proliferation before AC is started.^9^ In addition, response to NAC, unlike AC, is observable, so could potentially guide subsequent treatment decisions.

**CONTEXT**

**Key Objective**
To determine whether giving the first 6 weeks of chemotherapy preoperatively might safely reduce recurrence risk in patients with locally advanced but operable colon cancer.
**Knowledge Generated**
Neoadjuvant oxaliplatin-fluoropyrimidine chemotherapy for radiologically selected locally advanced operable colon cancer can be delivered safely and results in marked tumor down-staging, increased complete resection rates, and better disease control at 2 years than the same chemotherapy given postoperatively. Histologic tumor regression correlates strongly with freedom from recurrence. Less response was seen in mismatch repair (MMR)–deficient than in MMR-proficient cancers. Panitumumab did not enhance the benefit of neoadjuvant chemotherapy.
**Relevance *(A.H. Ko)***
A short course of neoadjuvant chemotherapy represents a reasonable treatment option for patients with clinical stage T3+ operable, MMR-proficient colon cancer. However, further study and validation are required before adopting this as the preferred standard of care over up-front surgical resection.*
*Relevance section written by *JCO* Associate Editor Andrew H. Ko, MD, FASCO.

However, there are potential disadvantages of NAC that have delayed its evaluation in colon cancer. Might toxicity during NAC compromise fitness for surgery or increase perioperative complications? Might chemoresistant cancers progress during NAC reducing the chance of surgical cure? Given the imprecision of radiologic staging, might low-risk patients be exposed to the toxicities and inconvenience of chemotherapy when surgery alone might have been considered sufficient?

By 2008, oxaliplatin-fluoropyrimidine combinations had demonstrated good efficacy and tolerability as both adjuvant and advanced disease treatment,^3,10^ and spiral computed tomography (CT) scans could more reliably identify moderate- to high-risk colon cancers.^11^ We therefore initiated Fluorouracil, Oxaliplatin and Targeted Receptor pre-Operative Therapy (FOxTROT),^12^ a randomized trial assessing the benefits and risks of advancing part of standard AC into the NAC setting. A short duration of NAC—just 6 weeks—was chosen to minimize the risk of on-treatment progression or residual toxicity.^10,13^ The planned total duration of chemotherapy was equal in both arms, allowing evaluation of sequencing rather than duration. An optional subrandomization evaluated whether, as in *RAS*-wildtype (wt) metastatic disease,^12,14,15^ adding panitumumab enhances response to NAC. An internal safety and feasibility pilot was included.^12^

## METHODS

### Trial Procedures

The initial factorial (2 × 2) design randomly assigned participants 2:1 to NAC-surgery-AC (NAC group) versus surgery-AC (control group), with *RAS*-wt tumors also randomly assigned 1:1 to receive panitumumab or not during the first 6 weeks of chemotherapy. From June 2011, with the addition of cetuximab to AC having proven ineffective,^16,17^ the panitumumab subrandomization was restricted to the NAC group. In 2014, with ongoing international studies examining 12-week AC,^4^ a protocol modification allowed the option of a shorter 12-week chemotherapy duration (6-week NAC + 6-week AC *v* 12-week AC) in lower-risk or older patients.

The eligibility criteria included biopsy-confirmed colon cancer, CT-predicted T3-4 with extramural extension ≥ 5 mm (modified to ≥ 1 mm after the pilot phase), M0, and being fit for both surgery and chemotherapy. Patients with bowel obstruction were eligible if first defunctioned with a stoma.

Treatment allocations were done by telephone or internet, using a minimized random assignment procedure balancing for age, CT-predicted T stage and N stage (T3/T4; Nx/N0/N1/N2), site of tumor, chosen chemotherapy (oxaliplatin-fluorouracil [OxFU]/OxCap), and defunctioning colostomy (yes/no).

National and institutional approvals were obtained for the Protocol (online only). Patients provided written informed consent. An Independent Data Monitoring Committee reviewed the accumulating data yearly.

### Treatment

OxFU was given using a modified FOLFOX schedule^13^: oxaliplatin 85 mg/m^2^ plus *l-*folinic acid 175 mg 2-hour infusion, fluorouracil 400 mg/m^2^ bolus, 2,400 mg/m^2^ 46-hour infusion, repeated once every 2 weeks. A total of 24 (or, optionally, 12) weeks' treatment was planned (3 NAC cycles + 9 [or 3] AC; or 12 [or 6] AC). Dose reductions, treatment delays, and early cessation for toxicity were permissible as in routine practice.

Panitumumab, if allocated, was infused at 6 mg/kg over 30-90 minutes before each of the first three cycles of OxFU. If randomization for panitumumab was not planned, OxCap^18^ could be used instead of OxFU: oxaliplatin 130 mg/m^2^ 1-hour IV infusion day 1, then oral capecitabine 1,000 mg/m^2^ twice a day days 1-14, repeated 3-weekly (2 cycles NAC + 6 [or, optionally, 2] AC; or 8 [or 4] AC).

Surgery was scheduled 4-6 weeks after completing NAC or, for control group patients, as soon as possible after random assignment. AC was scheduled to start 6-10 weeks after surgery regardless of the histologic stage at resection. Despite this intent, if chemotherapy was not given, reasons for its omission were recorded. Clinical follow-up was according to routine practice except that, to identify any recurrent disease for the primary outcome, a full clinical assessment (including carcinoembryonic antigen and a pelvis/thorax/abdomen CT scan) at 2 years after random assignment was mandatory. Six-monthly carcinoembryonic antigen and yearly abdominal CT scans were recommended for the first 3 years.

### Radiologic and Pathologic Staging

Lead histopathologists, radiologists, surgeons, and oncologists were appointed at each center. Radiology and histopathology training sessions were held, reaching over 300 consultants. Analyses are based on local radiologists' and histopathologists' reports, with an additional central review of regression grade, blinded to treatment allocation and outcome.

### Outcome Measures

The primary outcome was residual or recurrent disease within 2 years from random assignment. This was chosen to maximize statistical power, as chemotherapy effects on recurrence are concentrated in this period.^19^ Residual disease was defined as no resection, or macrosocopic incomplete resection (ie, residual tumor or metastases) after surgery but did not include those classified as R1 or R2 on pathologic review. To avoid lead-time bias, patients who did not have curative resections were classified as having the residual disease on day one after random assignment. Short-term efficacy was assessed by the rate of complete resection (R0) versus incomplete (R1, R2) or no resection, pathologic tumor, nodes, metastases stage (version 5), extramural venous invasion (EMVI), depth of invasion beyond muscularis propria, and Dworak tumor regression grade.^20^ The primary outcome for the panitumumab subrandomization was the depth of extramural spread. Adverse effects of chemotherapy (CTCAE V3.0) and perioperative morbidity were recorded on case record forms. Recurrence and survival status was updated annually. In addition, dates and causes of death were obtained through national registries.

### Statistical Methods

FOxTROT aimed to randomly assign 1,050 patients to detect a 25% proportional reduction in 2-year recurrence with NAC (eg, 32% reduced to 24%) with 80% power at *P* < .05. Log-rank, intention-to-treat analyses, including all randomly assigned patients and ignoring panitumumab allocation, were used to assess the statistical significance of differences in event rates. Some of the scheduled 2-year after random assignment scans were done later than 2 years, and it was assumed that recurrences (12 NAC, six control) detected on late (median 26 [IQR, 8-70] days later) scans would have been detected if the scan had been undertaken on day 730 as scheduled. Results with and without this assumption were similar (Data Supplement, online only). Deaths from noncolorectal cancer causes without recorded recurrence were treated as censoring events, ie, not counted as primary outcomes. T tests and Mantel-Haenszel tests of association used SAS 9.4 software (SAS Institute, Cary, NC). Planned subgroup analyses were of NAC efficacy by randomization stratification variables and by biomarkers potentially predictive of treatment efficacy.

## RESULTS

### Characteristics of the Patients

Between May 15, 2008, and December 23, 2016, 1,053 (41%) of 2,591 potentially eligible patients were randomly assigned 2:1 to the NAC group (n = 699) or control group (n = 354), from 85 centers: 79 in the the United Kingdom (n = 949), three in Denmark (n = 88), and three in Sweden (n = 16). Baseline characteristics were balanced across groups (Data Supplement). Chosen chemotherapy was OxFU in 756 (72%), OxCap in 297 (28%); the planned total chemotherapy duration was 24 weeks in 992 (94%), and 12 weeks in 61 (6%). A total of 279 patients participated in the NAC ± panitumumab subrandomization. The median age was 63 years; baseline CT suggested T4 disease in 268 (25%) and lymph-node involvement in 792 (75%). The median follow-up was 3.1 (IQR, 2.5-4.9) years.

### Treatment Delivery

**FIG 1.**
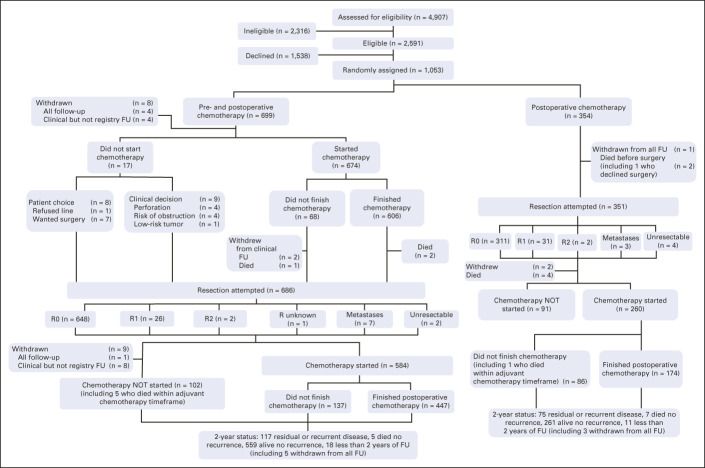
CONSORT diagram showing flow of patients through the FOxTROT trial. FOxTROT, Fluorouracil, Oxaliplatin, and Targeted Receptor preoperative therapy.

Nine patients (eight NAC, one control) withdrew immediately after random assignment, providing no trial-specific follow-up. Of 691 allocated to NAC who provided clinical follow-up (Fig 1), 674 (97.5%) started NAC, at a median of 11 (IQR, 7-14) days after random assignment, and 606 (90%) completed the full 6-week course (Data Supplement); of 17 (2.5%) who did not start NAC, seven received AC, so 681 of 691 (98.6%) received chemotherapy at some point in their treatment. In total, 30 of 691 (4.3%) patients allocated to NAC developed symptoms of obstruction before, during, or after receiving NAC, of whom one died of stroke and 29 underwent primary tumor resection (five after stenting). Two NAC patients withdrew after chemotherapy and three died beforehand, so 686 of 689 (99.6%) patients with follow-up went for surgery. Of 354 control patients, one withdrew, two died before surgery, and 351 (99.2%) went for surgery at a median of 14 days (IQR, 9.0-20.0) after random assignment.

### Tolerability

There were, if anything, fewer serious perioperative complications after NAC than after immediate surgery (Table 1); fewer had anastomotic leaks or abdominal abscesses: 4.7% (32/682) versus 7.4% (26/350), *P* = .072. Fewer required emergency reoperation (4.3% [29] *v* 7.1% [25], *P* = .050), or suffered complications prolonging hospital stay: 11.6% (79) versus 14.3% (50), *P* = .21. Fewer deaths from noncolon cancer causes without recorded recurrence occurred within 2 years from random assignment: 4 (0.6%) of 699 NAC patients compared with 6 (1.7%) of 354 control patients (*P* = .076) (Data Supplement). Toxicities reported during NAC or AC were those expected with OxFU and OxCap chemotherapy (Data Supplement).

**TABLE 1.**
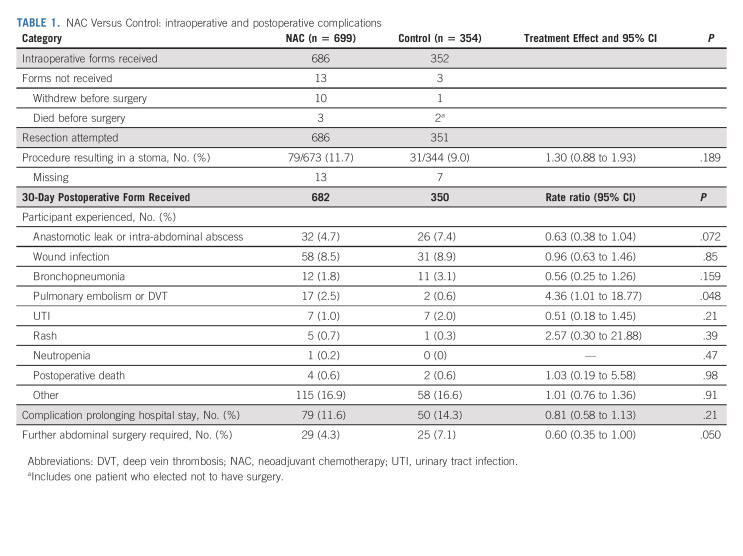
NAC Versus Control: intraoperative and postoperative complications

### Histopathologic Outcomes

**FIG 2.**
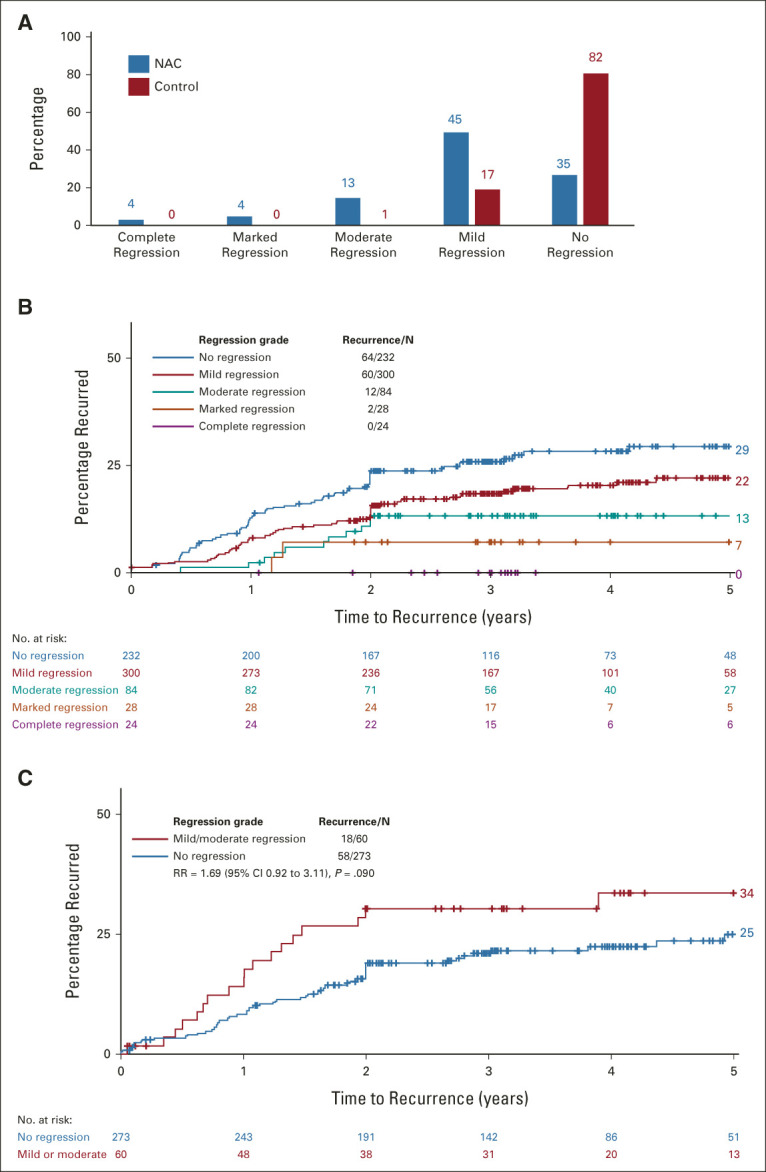
(A) Tumor regression grade by treatment allocation in the NAC group, and recurrence after surgery by regression grade in (B) NAC and (C) control groups. NAC, neoadjuvant chemotherapy; RR, rate ratio.

Substantial reductions in T stage, N stage, and EMVI were seen in the NAC group, compared with controls (Data Supplement). Notably, T4 disease was reduced from 107 of 351 (31%) in the control group to 142 of 686 (21%) after NAC, *P* < .001. More NAC than control patients had histopathologically complete (R0) resections: 94% (648/686) versus 89% (311/351), *P* < .001. Incomplete resection (R1, R2, or residual metastases) was reduced: 5.1% (35/686) compared with 10.3% (36/351), as was the risk of undergoing surgery with no attempted resection: 0.3% (2/686) versus 1.1% (4/351) (Data Supplement). Patients allocated to NAC also had significant reductions in a range of other measures of tumor dimension and nodal invasion, in particular, tumor regression grading: complete regression in 24 (4%), with marked, moderate, or mild regression in a further 412 (62%) patients. By contrast, 82% (273) allocated to control were scored by the central blinded review as showing no evidence of regression: Figure 2A and Data Supplement.

Of 351 control patients undergoing surgery, 83 (24%) did not meet standard criteria for AC out-with the trial^21^: nine were stage I (T ≤ 2, N0, M0), and 74 were low-risk stage II (T3, N0, M0, EMVI-negative, not high tumor grade or budding): Data Supplement.

### Postoperative Chemotherapy

Despite postoperative AC being recommended regardless of histology, AC was started more often in the NAC (584/686 [85%]) than control group (260/351 [74%]), at median postoperative intervals of 49 (IQR, 41-56) days and 48 (IQR, 42-56) days, respectively. In patients with histologically high-risk tumors (node-positive, T4 or EMVI), uptake of chemotherapy was similar in the NAC and control groups: 88% (344/390) versus 88% (223/253), *P* = .98. By contrast, patients with low-risk histology were much more likely to start AC if allocated to NAC than control (81% [240/296] *v* 39% [37/94], *P* < .0001, Data Supplement). There were five recurrences within 2 years among the 57 control patients with low-risk histology who did not receive AC.

### Recurrence and Survival

**FIG 3.**
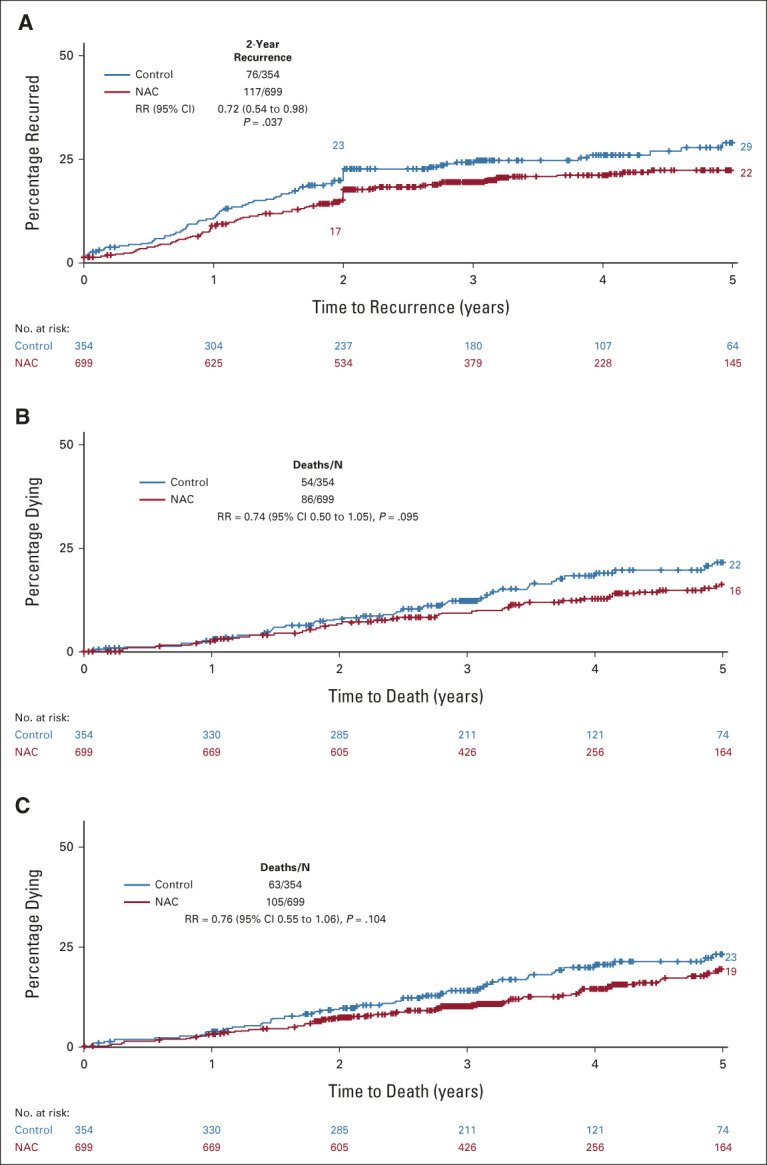
Risk of (A) recurrence or persistent disease, (B) death from colon cancer, and (C) death from any cause: NAC *v* control. NAC, neoadjuvant chemotherapy; RR, rate ratio.

The primary outcome, residual or recurrent disease within 2 years, occurred less often in NAC group patients: 16.9% (118/699) NAC versus 21.5% (76/354) control. This corresponded to a 28% lower recurrence rate with NAC than control: rate ratio (RR) = 0.72 (95% CI, 0.54 to 0.98, *P* = .037, Fig 3). The proportional reductions in colon cancer–specific mortality (RR = 0.74 [95% CI, 0.52 to 1.05, *P* = .095]), and all-cause mortality (RR = 0.76 [95% CI, 0.55 to 1.06, *P* = .104]) were of similar magnitude but did not reach statistical significance. There was no difference in death from noncolon cancer causes: 19 (2.7%) of 699 neoadjuvant chemotherapy (NAC) patients compared with 9 (2.5%) of 354 control patients (*P* = .87).

Risk of recurrence was strongly related to histologic regression grade (Fig 2B): 5-year recurrence was 30% for those with no regression, falling progressively to 22% in mild, 13% in moderate, 7% in marked, and 0% in complete regression categories. Conversely, in the control group (not receiving NAC), the recurrence rate for the few tumors with a histologic appearance scored as mild (58/333 [17%]) or moderate (2/333 [1%]) regression was, if anything, higher than in tumors with no regression (RR = 1.66 [95% CI, 0.91 to 3.03], *P* = .102, Fig 2C).

### Panitumumab

There was no indication that panitumumab enhanced the efficacy of NAC in RAS-wt patients: depth of extramural invasion was similar with versus without panitumumab: 6.2 versus 7.2 mm, *P* = .48. Seventeen percent (23/137) NAC + panitumumab versus 23% (30/133) NAC alone showed moderate or greater primary tumor regression (Data Supplement). The 2-year risk of residual or recurrent disease did not differ significantly: 18 versus 24 events; RR = 0.67 (95% CI, 0.36 to 1.23), *P* = .19, Data Supplement.

### Subgroup Analyses

**FIG 4.**
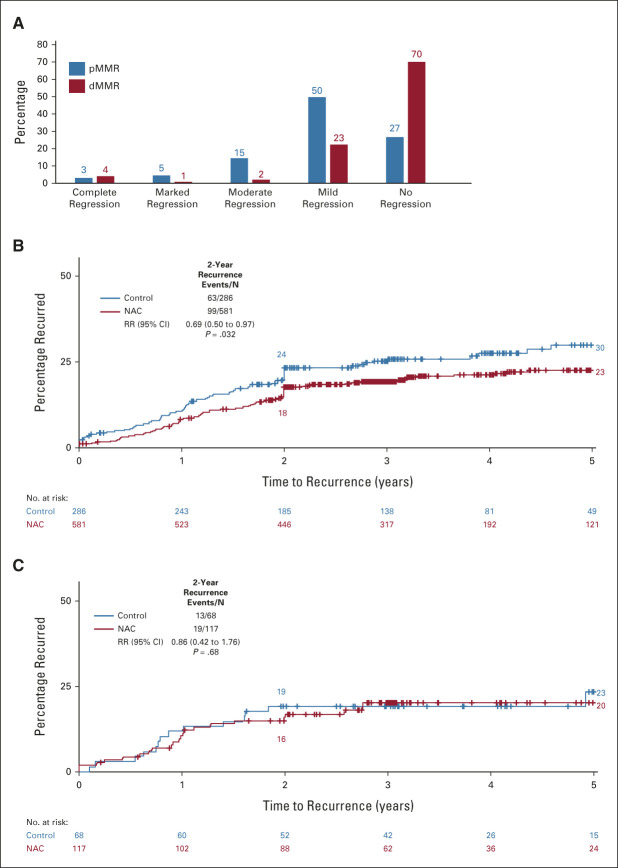
(A) Regresssion grade by MMR status in preoperative chemotherapy group and (B and C) recurrence or residual disease by treatment allocation for patients with pMMR or unknown MMR status^a^ tumors and for dMMR tumors. ^a^Seventy-six of five hundred fifty-three (14%) had unknown MMR status. dMMR, MMR-deficient; MMR, mismatch repair; pMMR, MMR-proficient; RR, rate ratio.

There was limited statistical power to investigate heterogeneity between subgroups in the effect of NAC on 2-year recurrence (Data Supplement). The more highly powered comparison of tumor regression rates within subgroups (Data Supplement) showed similar efficacy of NAC in different radiologic T and N stages, left- and right-sided tumors, age groups, sexes, with OxCap and OxFU, with shorter or longer proposed treatment duration, whether RAS-wt or mutant, and whether or not randomly assigned for panitumumab. The only strong association seen was with mismatch repair (MMR) status: moderate or greater regression after NAC was seen in just 7% (8/115) of MMR-deficient (dMMR) compared with 23% (128/553) of MMR-proficient (pMMR) tumors (*P* < .001): Figure 4A and Data Supplement. Similarly, there was no apparent reduction in 2-year disease recurrence in NAC patients with dMMR tumors (RR = 0.86 [0.42 to 1.76], *P* = .68), whereas, in pMMR tumors, the reduction in 2-year recurrence was significant: RR = 0.69 (0.50 to 0.97), *P* = .043: Figure 4. A similar pattern was seen for deaths from colon cancer (Data Supplement).

## DISCUSSION

FOxTROT is, to our knowledge, the first phase III trial evaluating NAC in operable colon cancer. It shows that short-course (6-week) NAC can be delivered safely and produces substantial tumor regression and downstaging, reducing the likelihood of incomplete resection. The primary objective—to detect a reduction in residual or recurrent disease within 2 years—was achieved, with a 28% lower event rate with NAC than AC. Chemotherapy toxicity was similar whether given before or after surgery, and surgical complications were, if anything, less in the NAC group.

FOxTROT results indicate that NAC then AC may be superior to conventional postoperative chemotherapy. Current clinical guidelines recommend preoperative chemotherapy as an option only for cT4 colon cancer because of low-quality evidence.^2,22^ It is therefore especially important to scrutinize the validity of the FOxTROT findings, and what guidance these provide for those considering implementing NAC.

Perhaps the first question is whether the observed reduction in 2-year residual or recurrent disease is sufficient to support a change in practice. This primary outcome was chosen because chemotherapy effects on recurrence are concentrated in the first 2 years,^19^ so we anticipated that any greater efficacy of NAC over AC would also be seen in this period, and this was precisely what we found. Also, most postoperative therapies that reduce early colorectal cancer recurrence also improve long-term survival.^19,23,24^ Hence, 3-year disease-free survival (DFS) is accepted as a basis to change practice. Unlike DFS, deaths from causes unrelated to cancer were not included in our primary outcome as, with both groups receiving chemotherapy, similar numbers were anticipated. Such deaths provide an important safety signal but are not a measure of efficacy.^25^ There were just 10 within 2 years and, reassuringly, fewer in the NAC than control group. So, had we used DFS rather than 2-year recurrence as primary outcome, the results would have been similar: RR, 0.69; 95% CI, 0.52 to 0.93; *P* = .014 (Data Supplement). So, also, would a crude comparison of proportions of patients disease-free at the 2-year time point (82.5% [577/699] NAC *v* 76.8% [272/354] control, *P* = .027).

FOxTROT was not powered to detect differences in overall recurrence, cancer-specific survival, and overall survival, though the proportional reductions in these longer-term outcomes were of similar magnitude and add to the clinical relevance of the reduction in 2-year recurrence. Longer follow-up of FOxTROT and meta-analysis with more recent studies^26-28^ may help determine whether these outcomes are also improved.

Another question is whether the observed effect might be attributable to less complete compliance in the control than NAC group. FOxTROT was designed to compare the same total quantity of chemotherapy in the two arms; however, 98% allocated to NAC started chemotherapy, whereas 25% of controls did not receive any chemotherapy. Most, predictably, had low-risk tumors of whom only five recurred within 2 years. Had they received chemotherapy, we estimate that one recurrence might have been prevented, which would make no material difference to the study findings. Hence, poor compliance with AC in lower-risk patients cannot account for the overall trial result.

The main disadvantage of NAC is that some patients with radiologically misclassified low-risk tumors receive unnecessary chemotherapy. In FOxTROT, 24% of control patients were found to have T3, N0 tumors without additional risk factors. For such patients whose recurrence risk is low, chemotherapy benefits are usually considered insufficient to justify the toxicity.^21^ Our ongoing review of FOxTROT radiology, and advances in radiologic techniques,^29^ should help improve the risk stratification algorithm for the deselection of patients with these lowest‐risk cancers.

It should also be noted that overtreatment occurs with adjuvant chemotherapy as well as NAC: even for high-risk operable colon cancer, around 10 patients are treated to prevent one recurrence. Regression grade after NAC might be useful in identifying which patients do and do not benefit from chemotherapy. For example, AC may not be needed for those with marked histologic regression after NAC who have a low risk of subsequent recurrence. This would need rigorous evaluation since it is also possible that patients with an excellent response to NAC are those who would benefit most from postoperative AC. Only randomized trials, perhaps incorporating circulating tumor DNA monitoring, can resolve these uncertainties.

Interestingly, apparent regression was associated with worse outcomes in control group patients. This may be because appearances similar to those associated with response to NAC are, in untreated patients, instead, indicative of high tumor stromal content, a poor prognostic feature.^30^ Since histologic regression is only prognostic after NAC, it must be an indicator of chemosensitivity, and not simply a surrogate for innately better-prognosis tumors. Hence, regression may reflect the effect of chemotherapy not just on the primary tumor but also on distant micrometastases and, consequently, long-term oncologic outcomes. This makes regression grade a potentially useful surrogate end point for future trials that might allow a more rapid assessment of novel NAC schedules.

FOxTROT, when launched in 2008, did not prespecify that lesser benefit was expected in dMMR tumors; however, dMMR has since been reported to predict nonbenefit from adjuvant fluoropyrimidine chemotherapy in colon cancer,^31^ and from NAC in gastroesophageal cancer.^32^ Consistent with these studies, tumor regression was far less common in dMMR than pMMR tumors, although four pathologic complete regressions were seen. No reduction in recurrence was seen in patients with dMMR tumors. FOxTROT has demonstrated the feasibility of rapid KRAS testing before the panitumumab random assignment, and clinicians considering using NAC could implement rapid MMR assessment of diagnostic biopsies to exclude dMMR patients for whom neoadjuvant immunotherapy seems a more promising option.^33^

We chose a short, 6-week neoadjuvant treatment to reduce the risk of toxicity impairing surgical fitness, and chemoresistant cancers progressing to inoperability. This choice appears justified: NAC was well tolerated, with high completion rates. Surgical morbidity appeared lower rather than higher, which may be due to tumor downsizing after NAC. Despite the fact that one third of NAC patients showed no discernible histologic regression, only 4% developed obstructive symptoms requiring expedited surgery, and all but one who did so were still able to undergo successful primary cancer resection. Therefore, pending randomized trials to explore longer durations, a 6-week NAC treatment remains our recommendation.

Over 80 hospitals took part in FOxTROT indicating that the results of this real world trial are generalizable to clinical practice. The median age of patients was 63, somewhat younger than in an unselected colon cancer population, but similar to that in pivotal AC trials. Just 28% (292/1,052) were aged over 70 years, compared with about 50% of incident cases, so they are likely to represent a fitter subset of older patients. However, it is still notable that they benefited at least as much from NAC as the younger patients.

In summary, patients with locally advanced but resectable colon cancer, selected using standard CT, may safely undergo 6 weeks of NAC before colon resection and then completion of AC. This does not increase perioperative morbidity, substantial tumor regression is achieved, and disease control is significantly better at 2 years than with the same chemotherapy given entirely postoperatively. NAC, like AC, is of less certain benefit in dMMR than pMMR cancers. Tumor regression after NAC is a strong predictor of lower postoperative recurrence risk and may provide a useful guide for later treatment. Six weeks of NAC should be considered as a treatment option for patients with locally advanced colon cancer.

## APPENDIX 1. Birmingham Clinical Trials Unit With Birmingham Surgical Trials Consortium

Laura Magill, Richard Gray, Kelly Handley, Adrian Wilcockson, Zoe Gray, Dominic Lancaster, James Brown, Andrew Palmer, Ladan Adie, Georgia Kennedy

**Clinical Trial Information**

NCT00647530

**Writing Committee**: Dion Morton, MD^1^; Matthew Seymour, MD^2^; Laura Magill, PhD^3^; Kelly Handley, PhD^3^; Gina Brown, MD^4^; David Ferry, MD^5^; James Glasbey, MD^1^; Bengt Glimelius, MD^6^; Andy Palmer^3^; Jenny Seligmann, MD^2^; Søren Laurberg, MD^7^; Keigo Murakami, MD^8^; Nick West, MD^8^; Philip Quirke, FMedSci^8^; and Richard Gray, MSc^9^

^1^University Hospital Birmingham, Birmingham, United Kingdom

^2^St James's University Hospital, Leeds, United Kingdom

^3^University of Birmingham Clinical Trials Unit, Birmingham, United Kingdom

^4^The Royal Marsden Hospital, London, United Kingdom

^5^Russell's Hall Hospital, Dudley, United Kingdom

^6^Uppsala University, Uppsala, Sweden

^7^Aarhus University, Aarhus, Denmark

^8^Division of Pathology and Data Analytics, School of Medicine, University of Leeds, Leeds, United Kingdom

^9^Clinical Trial Service Unit, University of Oxford, Oxford, United Kingdom

### Contributions

Fluorouracil, Oxaliplatin and Targeted Receptor pre-Operative Therapy trial was designed by Dion Morton, Richard Gray, Matthew Seymour, Laura Magill, Gina Brown, David Ferry, Philip Quirke, and Bryan Warren. Analyses were undertaken by Kelly Handley, Richard Gray, Dion Morton, and Laura Magill. Nick West, Bryan Warren and Philip Quirke developed the pathology protocol, delivered the training, and undertook central pathological review. Keigo Murakami undertook central review of the tumor regression grading. Gina Brown designed and led the training for radiologists and provided support for central review of X-rays. Keigo Murakami, Alice Westwood and Nick West reported the mismatch repair protein immunohistochemistry. RAS testing was coordinated by Susan Richman (Leeds) and Philippe Taniere (Birmingham). Dion Morton, Matthew Seymour and Richard Gray drafted the report and revised it with input from all writing committee members.

**Data Monitoring Committee**: T. Crosby, J. Olliff, R. Peto (Chair)

**Trial Management Committee**: Gina Brown, David Ferry, Bengt Glimelius, Richard Gray, Kelly Handley, Tariq Ismail, Søren Laurberg, Laura Magill, Dion Morton, Alf Oliver, Phil Quirke, Matthew Seymour, Nigel Scott, Jenny Seligman, Ian Swift, Bryan Warren, Nick West

**Trial Steering Committee**: J. Northover, M. Parmar (Chair), M. Slevin

**Fluorouracil, Oxaliplatin and Targeted Receptor pre-Operative Therapy Collaborators**

*Aalborg University Hospital—*Eld M., Holt G., Jensen D., Jensen F., Olesen B., Yilmaz* M., Andersen L.; *Aarhus University Hospital—*Dawar M., Emmertsen K., Garm Spendler* K., Hansen F., Husted-Madsen A., Jørgensen J., Kold-Petersen H., Kristensen E., Laurberg S., Løve U., Nerstrøm P., Rosenkilde M., Spangsperg I., Stribolt K., Strobel T., Ugianskis A.; *Arrowe Park Hospital, The Wirral—*Abgamu D., Day* N., Walsh C.; *Barnsley District General Hospital—*Bannister J., Furniss D., Morgan S., Walkington* L., Williams K., Yates S.; *Basingstoke and North Hampshire Hospital—*Arnold S., Branagan G., Cecil T., Ilesley I., Mohamed F., Mustajab A., O'Neil H., Rees* C., Smith J., Venkatasubramaniam A.; *Birmingham Heartlands Hospital—*Bowley D., Geh I., Goldstein M., Hendrickse* C., Kelly A., Kussaibati R., Langman G., McArthur D., Pallan A.; *Bradford Royal Infirmary—*Bradley C., Cockroft K., Conn* A., Davies J., Lowe A., Ostrowski J., Rehman S., Steward M.; *Bristol Royal Infirmary—*Ayre G., Brooks H., Burt C., Callaway M., Chandratreya N., Drury K., Falk S., Gray E., Hargreaves S., Longman R., Mason J., Melegh Z., Miller K., Philips S., Pollentine A., Pullyblank A., Roach H., Robinson T., Roe A., Shelley-Fraser G., Slack N., Thomas* M., Tiliet T., Vassallo K., Walther A., Wong N.; *Castle Hill Hospital, Hull—*Cast J., Dhadda A., Fuller C., Gunn J., Hartley J., Hunter I., Khan M., MacDonald A., O'Grady H., Roy* R., Tiam R., Sanni L.; *Charing Cross Hospital, London—*Ahmad R., Aldin Z., Blunt D., Buchanan G., Cleator* S., Cohen P., Dawson P., Gajapathy V., George N., Goldin R., Gujral D., Imseeh G., Joseph M., Kabuubi P., Limbu P., Lloyd J., Lowdell C., Ng-Cheng-Hin B., Oommen N., Osborn M., Paraskeva B., Petkar I., Ramesh A., Ramsey C., Riddle P., Smith J., Springall R., Vanree K., Ward D., Ziprin P.; *Chesterfield Royal Hospital—*Clenton S., Dewdney A., Euinton H., Furniss D., Gupta R., Pritchard K., Tarapowewalla D., Wilshaw* V.; *Christie Hospital, Manchester—*Anderson I., Armstrong G., Benbow E., Braun* M., Carlson G., Chakrabarty B., Chow S., Hill J., Jones D., Kasir D., Kushwaha R., Laasch H., Lees N., Marti F., McGrath S., Mescallado N., Mullamitha S., Pritchard S., Royle J., Saunders M., Slade D., Watson D., Westwood T.; *Clatterbridge Center For Oncology, The Wirral—*Anderson R., Artioukh D., Campbell F., Eardley N., Hughes M., Hughes P., Johnson M., McFaul C., McNicol F., Moss A., Palmer* D., Ramachandran I., Rooney P., Shabbir J., Shaker A., Shelouh A., Wall M., Yesildag P.; *Countess of Chester Hospital—*Abbott G., Hamid B., Prichard K., Vimalachandran* D.; *Cumberland Infirmary, Carlisle—*Berry J., Hinson F., Jehangir E., Maarouf Z., Nicoll* J., Wilkinson B., Young F.; *Derriford Hospital, Plymouth—*Adams C., Armstrong E., Bracey T., Denson J., Eastlake L., Fox B., Froud J., Jackson S., Oppong F., Sherriff* D., Shirley J., Sleigh K.; Diana, *Princess of Wales Hospital, Grimsby—*Kweka E., McAdam G., Newton M., Pearson H., Peters M., Rajendran T., Rehman K., Roy* R., Sasapu K., Sheth A., Sumbwanyambe N., Wallace T.; *Doncaster Royal Infirmary—*Khaira* M., Kurien G., Robinson J., Wadsley J., White D., Wilkinson N., Young R.; *Dorset County Hospital—*Bostanci G., D'arrigo C., Dega R., Flubacher M., Hogben K., Lamparelli M., Lawrence K., Lewis M., Mikel J., Orbell* J., Osborne R., Talwar A., Taylor P., Thomas T.; *George Eliot Hospital, Nuneaton—*Abdulkarim J., Gopalakrishnan K., Jadhav V., Klear P., Marimuthu K., Palit A., Piramanayagam B., Scott-Brown* M., Shatrughan S., Vallance K.; *Good Hope Hospital, Sutton Coldfield—*Baijal* S., Chapman M., Glaholm J., Kelly A., Nelson C., Singh R.; *Harrogate District Hospital—*Buxton A., Culverwell A., Darby A., Harrison J., Last* K., Leinhardt D., Mahon C., Mawhinney R., Scott D., Scullion D.; *Hospital Malarsjukhuset Eskilstuna—*Lind* P., Milosavljevic Z., Nordén A.; *Huddersfield Royal Infirmary—*Anwar S., Brown N., Cowley J., Dent* J., Gonsalves S., Gupta P., Hofmann U., Holdsworth P., Hyde J., Ilsley D., Knights R., Littleford S., Raja U., Roberts C., Wood T.; *Ipswich Hospital—*Chapman J., Crabtree M., Ng R., Orrell J., Sherwin* E., Smith S., Soomal R.; *Leighton Hospital, Crewe—*Adamson V., Braun* M., De A., Hardman J., Khan A., Nockolds C., Tee M.; *Macclesfield District General Hospital—*Khan U., Lavin V., Lawrence M., McBain C., Radharkrishna* G., Sil R., Tang A., Weerasinghe S., Wilkinson L.; *Manchester Royal Infirmary—*Gray G., Hill* J., Lee S., Wright P.; *Manor Hospital, Walsall—*Church R., Hartley A., Holland C., Howe R., Kunene* V., Marshall K., Muscroft T., Odogwu S., Ramaema D., Thompson A., Wagstaff L.; *Mount Vernon Hospital, Greater London—*Chiu M., Glynne-Jones* R., Goh V., Gupta A., Ilangovan R., Langner E., Livingstone J., Richman P., Russell J., Train M.; *Musgrove Park Hospital, Taunton—*Barlow* C., Burn P., Edwards T., Eyre-Brook I., Geraghty J., Karnati G., Mackey P., Taylor J., Walther J.; *New Cross Hospital, Wolverhampton—*Grumett* S., King M., Li P., Mangalika S., Mehra R., Qaiyum M., Rao A., Williams G.; *North Middlesex Hospital—*Borgstein R., Bridgewater* J., Firth J., Melville D., Ng M., Oluwajobi O., Rees J., Van Someren C.; *Northern Center for Cancer Treatment, Newcastle upon Tyne—*Coxon* F., Hainsworth P., Needham S., Scott J.; *Odense University Hospital—*Andersen I., Andersen P., Asmussen J., Hansen T., Hoffmann A., Jensen K., Madsen G., Pfeiffer* P., Salomon S., Thode Sorensen M.; *Pinderfields General Hospital, Wakefield—*Alkhaldi* A., Anathhanam A., Brittenden J., Fawole A., Jackson A., Jackson A., Kamposioras K., Kerr S., Kumaran G., Lupton S., Macklin C., Morrison C., Parchment-Smith C., Rogers M.; *Poole General Hospital—*Alexander J., Clarke A., Harinarayanan S., Harle* A., Heckford L., Hickish T., Lee-Elliot C., Leonard A., Martland G., Nash G., Qureshi T., Talbot R., Tarver D., Williams S., Wood A.; *Princess Alexandra Hospital, Harlow—*Bridgewater* J., Goonewardene T., Lalude O., Melcher L., Moe M., Partridge W., Sundaresan V., Vazquez I., Vivekanandan S.; *Queen Alexandra Hospital, Portsmouth—*Agrawal N., Atchley J., Beable R., Conti J., Cowlishaw D., Higginson A., Khan J., Marley N., McCartney J., Muthuramalingam* S., O'Leary D., O'Callaghan A., Pares D., Parvaiz A., Senapati A.; *Queen Elizabeth Hospital Birmingham—*Adedeji O., Bach S., Christensen J., Deshmukh N., Elbrønd H., Forde C., Gareja J., Hejmadi R., Iatrapoulos G., Ismail T., Jain A., Keh C., Levi F., McAvin L., McCafferty I., Middleton* G., Nygaard T., Page A., Radley S., Rafii S., Steven N., Suggett N., Taniere P., Tattersall D.; *Queen Elizabeth Hospital, Gateshead—*Devarajan G., Gulati M., Kerwat R., Maisey N., Mikhaeel* G.; *Queen's Hospital, Romford—*Ball S., Banerjee S., Bhargava A., Gershuny A., Gutmann J., Huang J., Igbokwe U., Johnston D., Raouf* S., Ray A., Saeed I., Tanner P., Wain M., Ward A., Wilson E.; *Queens Medical Center, Nottingham—*Acheson A., Arora A., Banerjea A., Dhingsa R., Dunn W., Hameed K., Ilyas M., Lopez Escola* C., Maxwell-Armstrong C., Mills J., Mohan V., Newcombe K., Potter V., Robinson M., Scholefield J., Walker G., Walter K., Williams J., Zaitoun A.; *Raigmore Hospital, Inverness—*Arnott S., Eason D., McPhail N., Mmeka* W., Stenhouse G., Walker K., Watson A., Whillis D.; *Randers Regional Hospital—*Andersen L., Cristensen J., Nygaard T., Spangsberg I.; *Regional Hospital Horsens—*Kristensen E., Madsen G., Rasmussen J.; Royal Derby Hospital*—*Chondra A., Kulkarni R., Lund* J., Menon S., Semeraro D., Singh R., Speake W., Tyriakidis K., Worth C.; *Royal Devon & Exeter Hospital—*Chandler I., Daniels I., Edwards D., Harries S., Osborne* M.; *Royal Free Hospital, London—*Bell J., Krell* D., Mayer A., Meaden L., Ogunbiyi O., Planche K., Watkins J.; *Royal Lancaster Infirmary—*Bronder C., Eaton* D., Mapstone N., Taylor A., Tibke C.; Royal *Marsden Hospital, London/Sutton—*Brown G., Cunningham* D., Hatt S., Tekkis P., Thomas J., Wotherspoon A.; *Royal Preston Hospital—*Dobson M., Lau S., Mitchell* P., Peristerakis I., Pitt M., Scott N., Susnerwala S.; *Royal Stoke University Hospital—*Adab F., Britton I., Clarke A., Dawson R., Farmer M., Ghiridaran* S., Hall C., Howitt C., Kalyanasundaram K., Kirby R., Latifaj B., Lengyel A., Parkinson K., Smith V.; *Royal United Hospital Bath—*Biddlestone L., Brydon-Hill R., Courtney E., Cox A., Dalton S., De Winton* E., Gangadhara S., Gibbs L., MacDonald K., Medley L., Meehan C., Phillips A., Williamson M.; *Russells Hall Hospital, Dudley—*Abbas H., Ashgar S., De Naurois J., Ferry D., Gerai S., Grumett* S., Hall A., Ibrahim A., Kaur J., Kawesha A., Maleki K., Momtahan N., Murukesh N., Patel R., Ramaiah S., Sherif A., Shinde V., Watts A., Youssef A.; *Salford Royal Hospital—*Burnett H., Hayes S., Soop* M., Vinod C.; *Salisbury District Hospital—*Agombar A., Attlee J., Branagan* G., Burroughs S., Chave H., Cook I., Cook S., Fuller C., Iveson T., McGee S., Shablak A., Sleight S., Watts C., Wheater M., Willis M.; *Sandwell and West Birmingham Hospitals—*Cruickshank N., Davies M., Joy H., Muzaffar S., Orme* A., Punia P., Rea D.; *Scunthorpe General Hospital—*Ahmad S., Coup A., Dent K., Hamid A., Moore P., Muzaffar Ahmad S., O'Toole* L., Pai D.; *Southampton General Hospital—*Armstrong L., Bateman A., Bateman* A., Beck N., Bennett J., Blaquiere R., Bradbury J., Breen D., Bryant T., Carr N., Chau C., Dudding T., Ellis S., Grierson C., King A., Knight J., Lane C., Nichols P., Smallwood J., Sommerlad M.; *Southend Hospital—*Chappell M., Dworkin M., Jain S., Malhotra A., Nichols L., Praveen B., Tsang* D.; *Southmead Hospital—*Hopkins K., Loveday E., Lyons* A., Rooney N., Santamaria M.; *Southport & Formby District General Hospital—*Ali N., Chatterjee* M., Chiphang A., Dundas S., Morris A., Sun Myint A., Zeiderman M.; *St George's Hospital, London—*Beddows E., Beharry N., Chong H., Chung E., Finlayson C., Greenhalgh R., Hagger R., Lofts* F., Melville D., Quarrell M., Ramwell A., Samol J.; *St James' University Hospital, Leeds—*Ambrose N., Anthoney A., Baker R., Baker H., Birbeck K., Botterill I., Burke D., Cairns A., Collinson F., Cooper R., Crossley A., Daverdede L., Dearden D., Finan P., Foltran L., Godfrey E., Grabsch H., Griffiths B., Guthrie A., Hall P., Hance J., Hyland R., Jayne D., Johnson R., Lambie H., Mekki R., Miskovic D., Rohatgi N., Rotimi O., Saunders R., Scott N., Seymour* M., Siller C., Sivakumar R., Swift S., Swinson D., Tolan D., Urquhart G., West N., Zubair S.; *St Mark's Hospital, Maidenhead—*Anyamene* N., Burling D., Kennedy R., Mahmud S., Moorghen M.; *Stepping Hill Hospital, Stockport—*Agrawal S., Gohar M., Hasan* J., Hodgkinson S., Howarth V., Lewinski M., Mehta S., Rai S., Saeed M.; S*vendborg Hospital—*Andersen P., Hoffmann A., Salomon S.; *The Royal Liverpool University Hospital—*Bermingham N., Campbell F., Carter P., Healey P., Heath R., Hughes M., Madi A., Montazeri A., Rooney* P., Smith D., Smith F.; *University College Hospital, London—*Ahmed M., Arkenau T., Hochhauser D., Obichere A., Rodriguez-Justo M., Shiu* K., Taylor S., Wong S.; *University Hospital Coventry—*Baragwanath P., Correa* P., James S., Shatwell W., Suotamo S., Williams N.; *University Hospital Lewisham—*Birch D., Brady* J., Byrne C., Hinsell J., Lanaspre E., Mikhaeel G., Roy R., Shah A., Singhera M.; *University Hospital of North Tees, Stockton—*Ahmad M., Garg D., Gill* T., Rao V., Wardle H., Wilson D.; *University Hospital of Wales—*Adams* R., Beehen R., Morgan M., Williams M.; *University Hospital Umeå—*Lindh B., Sjodin M.; *Uppsala University Hospital—*Ahlstrom H., Birgisson H., Dukic M., Folkesson J., Glimelius* B., Lidén A., Siilin H., Sköldberg F., Terman A.; *Velindre Hospital, Cardiff—*Adams* R., Bowden C., Hargest R., Horwood H., Jackson A., Morgan M., Owen R.; *Warrington Hospital—*Ford A., Gopal K., Madew R., Pranesh* N., Shareef D., Tighe M.; *Warwick Hospital—*Busby K., Correa* P., Flook M., Hartwell L., Patel R., Sanders S., Sinha R.; *West Middlesex University Hospital—*Ahmad* R., Desai S., Ramesh S., Swallow J.; *Weston General Hospital, Weston-Super-Mare—*Dymond H., Hilman* S., Lott M., O'Brien J., Radstone D., Sheffield E., Tomlinson M., West D.; *Weston Park Hospital, Sheffield—*Adam I., Alzouebi M., Amarnath T., Amin S., Balamurugan R., Chapple K., Coker O., Das T., Garikipati S., Genever A., Goodfellow P., Hampton J., Harris H., Hornbuckle* J., Kitsanta P., Kumar V., Martin J., Muzaffar M., Pledge S., Rao J., Rogers S., Svrinivasan D., Tiffin N., Waweru A., White T., Williams K.; *Wexham Park Hospital, Slough—*Ali M., Baxter S., Brown R., Charig M., De Souza F., Desai A., Gilbert J., Hadaki M., Hall* M., Harris C., Hassan A., Sharif H., Thyveetil M.; *Whittington Hospital, Londo*n*—*Arul D., Hochhauser D., Leonard* P., Mukhtar H., Murray D.; *Worcestershire Royal Hospital—*Baxter A., Churn* M., Farrugia D., Lake S., Roberts T., Smith G.; *Wrexham Maelor Hospital—*Bansal A., Bersassi A., Billings P., Chandran P., Corr C., Dixon R., Gollins* S., Kodavatiganti R., Pradeep K., Stockport J., Thornton M.; *Wythenshawe Hospital, Manchester—*Davenport A., McCartin F., Saunders* M., Sukumar S.; *Yeovil District Hospital—*Allison A., Bathurst N., Beaumont* E., Cooper E., Francis N., Hay M., Ockrim J., Perry J., Sephton M., Sparrow G.; *York Hospital—*Alexander D., Bandyopadhyay D., Bedford I., Chan S., Chintapatla S., Clarke A., Haselden J., Kell K., Last* K., Maughan N., Petty D., Stojkovic S., Woodcock N.; *Ysbyty Glan Clwyd, Rhyl—*Atkinson M., Evans S., Favill E., Gollins* S., Gupta M., Maw A., Rajagopal R.; *Ysbyty Gwynedd District General Hospital, Bangor—*Abdullah N., Bale* C., Bhalerao S., Fuller C., Lala A., Lord M., Saxton W.

*Represents the Local Principal Investigator.

## References

[1] GBD 2016 Disease and Injury Incidence and Prevalence Collaborators: Global, regional, and national incidence, prevalence, and years lived with disability for 328 diseases and injuries for 195 countries, 1990–2016: A systematic analysis for the Global Burden of Disease Study 2016. Lancet 390:1211-1259, 2017 28919117 10.1016/S0140-6736(17)32154-2 PMC5605509

[2] National Institute for Health and Care Excellence: Colorectal Cancer. NICE Guideline [NG151]. London, UK, 2020. https://www.nice.org.uk/guidance/ng151 32813481

[3] André T Boni C Mounedji-Boudiaf L : Oxaliplatin, fluorouracil, and leucovorin as adjuvant treatment for colon cancer. N Engl J Med 350:2343-2351, 2004 15175436 10.1056/NEJMoa032709

[4] Grothey A Sobrero AF Shields AF : Duration of adjuvant chemotherapy for stage III colon cancer. N Engl J Med 378:1177-1188, 2018 29590544 10.1056/NEJMoa1713709 PMC6426127

[5] Medical Research Council Oesophageal Cancer Working Party: Surgical resection with or without preoperative chemotherapy in oesophageal cancer: A randomised controlled trial. Lancet 359:1727-1733, 2002 12049861 10.1016/S0140-6736(02)08651-8

[6] Cunningham D Allum WH Stenning SP : For the MAGIC trial participants: Perioperative chemotherapy versus surgery alone for resectable gastroesophageal cancer. N Engl J Med 355:11-20, 2006 16822992 10.1056/NEJMoa055531

[7] Alieva M van Rheenen J Broekman MLD: Potential impact of invasive surgical procedures on primary tumor growth and metastasis. Clin Exp Metastasis 35:319-331, 2018 29728948 10.1007/s10585-018-9896-8 PMC6063335

[8] Hu Z Ding J Ma Z : Quantitative evidence for early metastatic seeding in colorectal cancer. Nat Genet 51:1113-1122, 2019 31209394 10.1038/s41588-019-0423-x PMC6982526

[9] Zeamari S Roos E Stewart FA: Tumour seeding in peritoneal wound sites in relation to growth-factor expression in early granulation tissue. Eur J Cancer 40:1431-1440, 2004 15177504 10.1016/j.ejca.2004.01.035

[10] Seymour MT Maughan TS Ledermann JA : Different strategies of sequential and combination chemotherapy for patients with poor prognosis advanced colorectal cancer (MRC FOCUS): A randomised controlled trial. Lancet 370:143-152, 2007 17630037 10.1016/S0140-6736(07)61087-3

[11] Smith NJ Bees N Barbachano Y : Pre-operative computed tomography staging of non-metastatic colon cancer predicts outcome: Implications for clinical trials. Br J Cancer 96:1030-1036, 2007 17353925 10.1038/sj.bjc.6603646 PMC2360118

[12] FOxTROT Collaborative Group: Feasibility of preoperative chemotherapy for locally advanced, operable colon cancer: The pilot phase of a randomised controlled trial. Lancet Oncol 13:1152-1160, 2012 23017669 10.1016/S1470-2045(12)70348-0 PMC3488188

[13] Cheeseman SL Joel S Chester JD : A "modified de Gramont" regimen of fluorouracil, alone (MdG) and with oxaliplatin (OxMdG), for advanced colorectal cancer. Br J Cancer 87:393-399, 2002 12177775 10.1038/sj.bjc.6600467 PMC2376131

[14] Maughan TS Adams RA Smith CG : Addition of cetuximab to oxaliplatin-based first-line combination chemotherapy for treatment of advanced colorectal cancer: Results of the randomised phase 3 MRC COIN trial. Lancet 377:2103-2114, 2011 21641636 10.1016/S0140-6736(11)60613-2 PMC3159415

[15] Douillard JY Siena S Cassidy J : Randomized, phase III trial of panitumumab with infusional fluorouracil, leucovorin, and oxaliplatin (FOLFOX4) versus FOLFOX4 alone as first-line treatment in patients with previously untreated metastatic colorectal cancer: The PRIME study. J Clin Oncol 28:4697-4705, 2010 20921465 10.1200/JCO.2009.27.4860

[16] Alberts SR Sargent DJ Nair S : Effect of oxaliplatin, fluorouracil, and leucovorin with or without cetuximab on survival among patients with resected stage III colon cancer—A randomized trial. JAMA 307:1383-1393, 2012 22474202 10.1001/jama.2012.385 PMC3442260

[17] Taieb J Tabernero J Mini E : Oxaliplatin, fluorouracil, and leucovorin with or without cetuximab in patients with resected stage III colon cancer (PETACC-8): An open-label, randomised phase 3 trial. Lancet Oncol 15:862-873, 2014 24928083 10.1016/S1470-2045(14)70227-X

[18] Schmoll H-J Tabernero J Maroun J : Capecitabine plus oxaliplatin compared with fluorouracil/folinic acid as adjuvant therapy for stage III colon cancer: Final results of the NO16968 randomized controlled phase III trial. J Clin Oncol 33:3733-3740, 2015 26324362 10.1200/JCO.2015.60.9107

[19] QUASAR Collaborative Group: Adjuvant chemotherapy versus observation in patients with colorectal cancer: A randomised study. Lancet 370:2020-2029, 2007 18083404 10.1016/S0140-6736(07)61866-2

[20] Dworak O Keilholtz L Hoffmann A: Pathological features of rectal cancer after preoperative radiochemotherapy. Int J Colorectal Dis 12:19-23, 1997 9112145 10.1007/s003840050072

[21] Baxter NN Kennedy EB Bergsland E : Adjuvant therapy for stage II colon cancer: ASCO guideline update. J Clin Oncol 40:892-910, 2021 34936379 10.1200/JCO.21.02538

[22] National Comprehensive Cancer Network: NCCN clinical practice guidelines in oncology: Colon cancer, version 2.2022. https://www.nccn.org/professionals/physician_gls/pdf/colon.pdf 10.6004/jnccn.2022.0008 35130500

[23] Sargent DJ Wieand HS Haller DG : Disease-free survival versus overall survival as a primary end point for adjuvant colon cancer studies: Individual patient data from 20,898 patients on 18 randomized trials. J Clin Oncol 23:8664-8670, 2005 16260700 10.1200/JCO.2005.01.6071

[24] André T Boni C Navarro M : Improved overall survival with oxaliplatin, fluorouracil, and leucovorin as adjuvant treatment in stage II or III colon cancer in the MOSAIC trial. J Clin Oncol 27:3109-3116, 2009 19451431 10.1200/JCO.2008.20.6771

[25] Cuzick J: Primary endpoints for randomised trials of cancer therapy. Lancet 371:2156-2158, 2008 18586160 10.1016/S0140-6736(08)60933-2

[26] Karoui M Gallois C Piessen G : Does neoadjuvant FOLFOX chemotherapy improve the prognosis of high-risk stage II and III colon cancers? Three years’ follow-up results of the PRODIGE22 phase II randomized multicentre trial. Colorectal Dis 23:1357-1369, 2021 33580623 10.1111/codi.15585

[27] Hu H Huang M Li Y : Perioperative chemotherapy with mFOLFOX6 or CAPOX for patients with locally advanced colon cancer (OPTICAL): A multicenter, randomized, phase 3 trial. J Clin Oncol 40, 2022 (supp 16; abstr 3500)

[28] Liu F Tong T Huang D : CapeOX perioperative chemotherapy versus postoperative chemotherapy for locally advanced resectable colon cancer: Protocol for a two period randomised controlled phase III trial. BMJ Open 9:e017637, 2019 10.1136/bmjopen-2017-017637 PMC6352769 30700474

[29] Nerad E Lahaye MJ Maas M : Diagnostic accuracy of CT for local staging of colon cancer: A systematic review and meta-analysis. Am J Roentgenol 207:984-995, 2016 27490941 10.2214/AJR.15.15785

[30] Hutchins GGA Treanor D Wright A : Intratumoral stromal morphometry predicts disease recurrence but not response to 5-fluorouracil-results from the QUASAR trial of colorectal cancer. Histopathology 72:391-404, 2018 28746977 10.1111/his.13326

[31] Sargent DJ Marsoni S Monges G : Defective mismatch repair as a predictive marker for lack of efficacy of fluorouracil-based adjuvant therapy in colon cancer. J Clin Oncol 28:3219-3226, 2010 20498393 10.1200/JCO.2009.27.1825 PMC2903323

[32] Pietrantonio F Miceli R Raimondi A : Individual patient data meta-analysis of the value of microsatellite instability as a biomarker in gastric cancer. J Clin Oncol 37:3392-3400, 2019 31513484 10.1200/JCO.19.01124

[33] Chalabi M Fanchi LF Dijkstra KK : Neoadjuvant immunotherapy leads to pathological responses in MMR-proficient and MMR-deficient early-stage colon cancers. Nat Med 26:566-576, 2020 32251400 10.1038/s41591-020-0805-8

